# Bed-to-Bed Transfer Program Among Patients Who Need Hospitalization in a Crowded Emergency Department in Taiwan

**DOI:** 10.34172/ijhpm.2021.88

**Published:** 2021-08-24

**Authors:** Nai-Wen Ku, Chu-Lin Tsai, Shyr-Chyr Chen, Chien-Hua Huang, Cheng-Chung Fang, Shiow-Ching Shun

**Affiliations:** ^1^School of Nursing, National Taiwan University, Taipei, Taiwan.; ^2^Department of Emergency, National Taiwan University Hospital, Taipei, Taiwan.; ^3^Department of Emergency Medicine, College of Medicine, National Taiwan University, Taipei, Taiwan.; ^4^Institute of Clinical Nursing, College of Nursing, National Yang Ming Chiao Tung University, Taipei, Taiwan.

**Keywords:** Crowding, Emergency Department, Length of Stay, Transfer Program

## Abstract

**Background:** Emergency department (ED) crowding is a universal issue. In Taiwan, patients with common medical problems prefer to visit ED of medical centers, resulting in overcrowding. Thus, a bed-to-bed transfer program has been implemented since 2014. However, there was few studies that compared clinical outcomes among patients who choose to stay in medical centers to those being transferred to regional hospitals. The aim of this study was to explore the transfer rate, delineate the factors related to patient transfer, and clarify the influence upon the program outcomes.

**Methods:** A retrospective cohort study was conducted using demographic and clinical disease factors from the patient electronic referral system, electronic medical records (EMRs) of a medical center in Taipei, and response to referrals from regional hospitals. The study included adult patients who were assessed as appropriate for transfer in 2016. We analyzed the outcomes (length of stay and mortality rate) between the referrals were accepted and refused using propensity score matching.

**Results:** Of the 1759 patients eligible for transfer to regional hospitals, 420 patients (24%) accepted the referral. Medical records were obtained from the regional hospitals for 283 patients (67%). After propensity score matching, the results showed that interhospital transfer resulted in similar median total length of stay (8.7 days in the medical center vs 7.9 days in regional hospitals; *P*=.245). In-hospital mortality was low for both groups (3.1% in the medical center vs 1.3% in regional hospitals; *P*=.344).

**Conclusion:** Transfer from an overcrowded ED in a medical center to regional hospitals in eligible patients results in non-significant outcome of total length of stay. With the caveat of an underpowered sample, we did not find statistically significant differences in in-hospital mortality. This healthcare delivery model may be used in other cities facing similar problems of ED overcrowding.

## Background

**Key Messages**

** Implications for policy makers**

- This transfer program is secure and feasible and may be used in other cities facing similar problems of emergency department (ED) overcrowding.
- The bed-to-bed transfer program could reduce time spent in the ED for patients and would not increase the total length of stay in hospital.
- After evaluating patients, physicians and nurse practitioners can make suggestions earlier in the patient care process and assist in transfers, thereby decreasing the ED length of stay for patients.

** Implications for the public**

Emergency department (ED) crowding, especially in medical centers, is a universal and important issue in the world. However, shortage of admission beds in the general wards for patients with common medical problems has a great effect on patient safety and contributes to worse patient outcomes when EDs become overcrowded. Patients and their families hesitate to transfer to regional hospitals because of concerns related to inferior quality of care and worse outcomes. This study found that the bed-to-bed transfer program was safe and resulted in similar outcomes between patients who were transferred and those who were not transferred. We hope this study will help to increase the public’s confidence in receiving treatments from regional hospitals for common medical problems. This will avoid overcrowding of the medical center ED and improve patient safety and quality of patient care.

Emergency department (ED) crowding is a universal and important issue in the world because it has a great effect on patient safety and contributes to worse patient outcomes.^1^ ED crowding not only lowers medical quality^2-4^ resulting in system overloading^5,6^ but also increases emergency boarding time that will ultimately decrease the capacity of the ED and cause medical treatment delays.^7,8^ This results in longer patient stays in the ED and is more likely to result in worsening of patient conditions and more intensive care unit (ICU) admissions.^9^ In addition, it raises the likelihood of in-hospital cardiac arrest and, thus, increases mortality rate.^10^

 Taiwan’s implementation of National Health Insurance starting in 1995 has changed patients’ medical utilization habits.^11^ Patients nowadays prefer to visit EDs in medical centers than to visit regional hospitals for trivial problems with lower severity levels. The situation invariably causes overcrowding and prolonged time spent in EDs.^12^ To improve this situation, the bed-to-bed transfer program has been implemented since February 2014. The purpose of the bed-to-bed transfer program is to transfer patients who needed hospitalization directly to regional hospital wards from the ED of medical centers in order to mitigate ED crowding, to increase emergency capacity, and to decrease time spent in the ED. Furthermore, patients can receive appropriate medical resources and care, and hopefully have a better outcome.

 According to this program, patients waiting for admission were eligible to be transferred from the medical center ED to a general ward in a regional hospital following an evaluation by medical personnel (ie, attending physicians or nurse practitioners). This was considered a nonurgent referral. This program has received positive feedback from patients who were satisfied with the referral procedure, medical procedure, patient safety and security, and service attitude.^13^ It was a different transfer model from that used in other studies, which often required transfer and further medical treatment as a result of severe diseases.^14-17^ In Taiwan, there are 19 medical centers and 83 regional hospitals. Generally, patients hesitate to visit regional hospitals because of concerns related to inferior quality of care and worse outcomes,^11,13,18^ but healthcare providers usually assume that patients who undergo transfer can receive suitable care in regional hospitals. However, there is little empirical evidence to support this assumption. We therefore sought to examine the outcomes for this program in Taiwan.

 Previous studies on transfer programs have shown that demographic factors, clinical disease factors, and ED length of stay influenced the length of hospital stay and mortality.^14-17,19^ However, it remained unclear how these factors are related to patient preference to transfer or whether patient transfer would result in different outcomes in terms of mortality and total hospital length of stay. A previous transfer study showed that 18% patients who were transferred to the regional hospitals suffered complications(eg, hospital-acquired pneumonia**)**.^20^ In contrast, one systematic review analyzing patient transfers from EDs in various hospitals found that there was no difference in mortality among transferred and non-transferred patients from medical, surgical, or EDs.^14^ Whether or not patients who undergo interhospital transfer would receive equal quality of care remains a controversial issue.

 Therefore, we hypothesized that transferring patients would not cause differences in the total length of stay or mortality rate compared with patients who were not transferred. The aim of this study was to explore the transfer rate, delineate the factors related to patient transfer, and clarify the influence upon outcomes (ie, total hospital length of stay and mortality rate).

## Materials and Methods

###  Study Design and Data Collection

 We conducted a retrospective cohort study, utilizing data from the emergency patient electronic referral system, Mars, electronic medical records (EMRs) from one medical center in Taipei, and hard copies of referral sheets from receiving regional hospitals. Mars, an emergency referral platform, contains information about patient basic data, major diagnoses, time of transfer leaving and arriving, and vital signs in Taiwan. The EMR provided patient demographic information and the Taiwan triage and acuity scale (TTAS), which evaluated a patient’s symptoms and severity, initial vital signs on triage, physical examination, and laboratory data in the ED. We manually extracted data from both electronic databases and performed quality assurance and control. In addition, we excluded patients who did not have information on discharge disposition, outcome, or referral sheets from regional hospitals.

###  Population Selection

**Figure 1:**
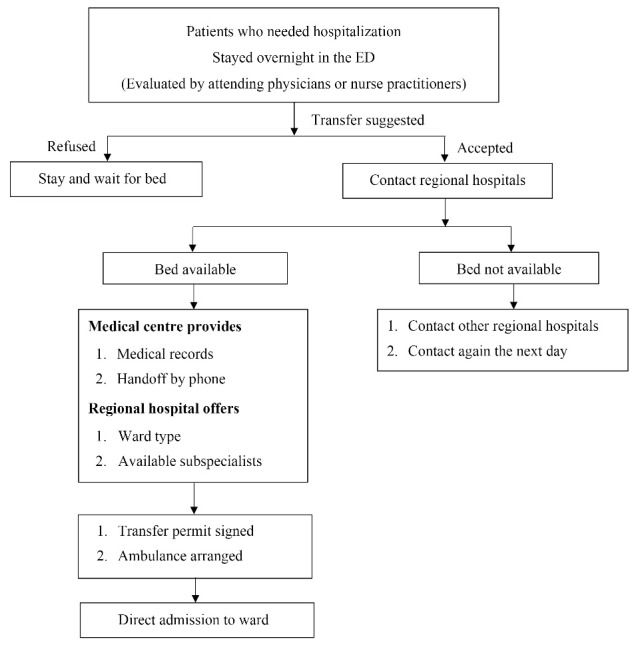
Transfer Process. Abbreviation: ED, emergency department.

 The study included all patients considered candidates for transfer from the medical center ED to regional hospitals from January 1, 2016, to December 31, 2016. A transfer process of bed-to-bed program is displayed in Figure 1. In brief, when a patient stayed overnight in the ED and was deemed to need hospitalization by attending physicians and/or nurse practitioners the next morning, a case manager would approach the patient for enrollment in the program. Exclusion criteria were patients younger than 20 years of age, those transferred to a nonregional hospital, those with psychosis as a major diagnosis, and those transferred for surgery or hospice care.

###  Study Variable

 We selected sets of variables a priori and examined their associations with the outcomes of the patient transferred. The data were collected with two electronic databases including the following items: patient age, gender, level of education, residential area, comorbidities measured by the Charlson Comorbidity Index (CCI), coronary artery disease status, hypertension status, bedridden status, history of tracheostomy, major surgery or illness status, and the presence of a do-not-resuscitate (DNR) order.

###  Clinical Characteristics

 We hypothesized that patient level, the 5-level triage rating, and transfer variables would influence outcomes. Variables of the ED that were included are as follows: TTAS data, vital signs, symptoms in triage, examinations after suggestions to transfer (eg, ultrasonography, computed tomography, endoscopy), initial and discharge diagnoses, and changes in diagnosis.

###  Transferred of Time Situation

 This study also analyzed time variables to clarify if time factors influenced outcomes. Time from triage to transfer suggestion included the time duration from triage to the moment it was suggested that the patient be transferred to a regional hospital. ED length of stay was the time from triage to ED discharge.

###  Outcome Measures

 Data were merged from Mars and the EMR by patient identification chart number. Outcomes of interest included mortality and total length of stay. Mortality indicated those patients who died while in the hospital, and total length of stay indicated the duration from medical center ED triage to discharge from the hospital (medical center or regional hospital).

###  Data Analysis

 Data were entered and analyzed using IBM SPSS statistics, version 24.0. Descriptive statistics were used to report the mean and standard deviation of the continuous variables; categorical variables were presented with counts and percentage. In univariate analyses, normally distributed data were compared using the independent *t* test and categorical data were compared using chi-square analyses. For all time-related variables in our study, their distribution was right-skewed, so we reported medians/interquartile ranges and performed non-parametric statistical analysis. Non-parametric confidence intervals were obtained by performing the bootstrapping procedure 1000 times. A two-sided alpha level of.05 was used for all statistical testing. A *P* value less than.05 was considered statistically significant with correction for multiple comparison.

 Propensity score matching method was used to balance baseline characteristics between the two groups.^21,22^ Factors related to transfer decision and outcomes of interest (eg, age, gender, CCI, Triage levels, Diagnosis) were included in a logistic regression model to predict the probability of transfer, namely, the propensity score for transfer^23-26^ (Table S1, Supplementary file 1). The propensity was then assigned to each individual. Transferred patients were matched to controls (non-transferred) one-to-one by a greedy nearest neighbor matching algorithm and matching without replacement, with a minimum match caliper of 0.0228.^27,28^ Numeric and graphic diagnostics were performed to check the balance of the matched pairs, including standardized mean difference (SMD) and distribution of the propensity scores.^29^ We used R3.5.3 software; statistics of the match were evaluated by the distribution of propensity score and SMD.^21,30^ As opposed to the traditional statistical test of a hypothesis, SMD greater than 0.25 was used as the indicator of distribution unbalance to avoid potential bias caused by sample size.^30^

## Results

**Figure 2:**
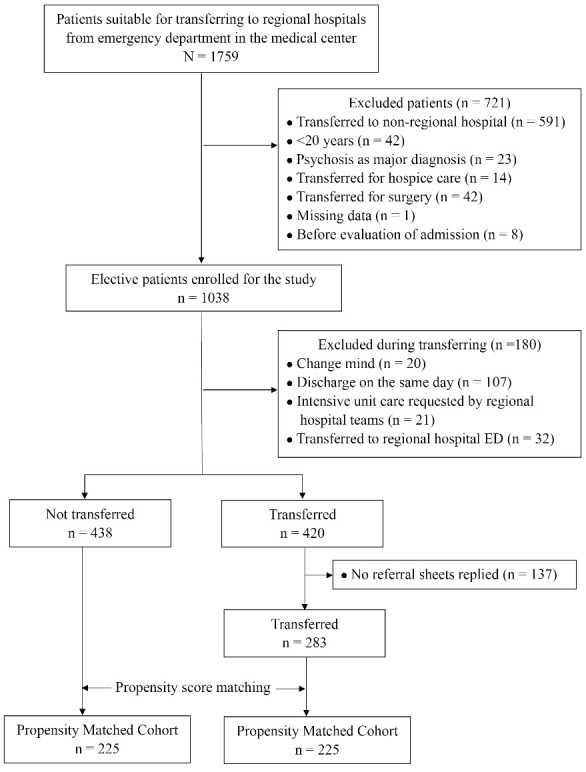
Patient Selection Algorithm. Abbreviation: ED, emergency department.

 A patient selection diagram is displayed in Figure 2. We examined a total of 1759 patients who were eligible for transfer from the medical center to regional hospitals. We excluded 901 transferred patients who were younger than 20 years of age, those transferred to a nonregional hospital, patients with psychosis as major diagnosis, patients who were transferred for surgery or hospice care, or patients who experienced a problem (eg, change mind, ICU requested by regional hospital teams) during the transfer. A total of 21 patients, in which ICU service was requested by regional hospital teams, were eventually transferred to the ICU of regional hospitals. Thus, they were excluded as the transfer program only considered patients suitable for care on the ward. Finally, a total of 721 patients were analyzed in this study. Of those, 420 (24%) underwent an interhospital transfer, and copies of the 283 patients’ responses to referral were received by regional hospitals (response rate 67%). There were no differences in demographic features among those whose referral sheets were replied vs. those whose referral sheets not replied (Table S2, Supplementary file 1). A total of 225 paired patients were included after propensity score matching.

 In the original unmatched cohort, younger patients were more likely to be transferred (mean age ± SD, 64.0 ± 19 vs 58.9 ± 19.7 years; *P* < .001). The presence of a DNR order was less likely among those who underwent transfer (4.8% vs 0.7%; *P* =.002). For Non-transfer or transferred patients who had a DNR order, their CCI scores were similar (mean ± SD 2.95 ± 2.1 vs 4.5 ± 6.4,* P* = .791). The transfer suggestion was made later in the non-transferred group with a longer time from triage to transfer suggestion (median 21.8 vs 17.7 hours, *P* < .001). There was no difference in residential area and CCI among those receiving transfers vs those who did not (Table 1). The 438 non-transferred patients spent a median of 28 hours boarding in the ED (from suggestion to transfer to departure from the ED); of them, 127 were treated in the ED and eventually were discharged. These 127 patients spent a median of 67.2 hours in the ED.

**Table 1:** Demographic, Medical Histories, Clinical Characteristics, and Time of Transfer of Patients Transferred and Not Transferred to Regional Hospitals Before Propensity Score Matching

	**Not Transferred (n = 438)**	**Transferred (n = 283)**	***P*** **Value** ^a^
**Demographic variables, No. (%)**			
Age (≥65 y)	226 (51.6)	113 (39.9)	**.002**
Gender (male)	231 (52.7)	152 (53.7)	.799
Education (above senior high school)	248 (56.6)	173 (61.1)	.230
Residential area			.980
Taipei city	206 (47.0)	135 (47.7)	
New Taipei city	203 (46.3)	130 (45.9)	
Other	29 (6.6)	18 (6.4)	
CCI, (mean ± SD)	1.4 ± 1.8	1.3 ± 1.9	.465
**Medical histories not included in CCI, No. (%)**			
CAD/valvular heart disease/arrhythmia	88 (20.1)	51 (18.0)	.491
Bedridden	16 (3.7)	4 (1.4)	.102
Tracheostomy	4 (0.9)	1 (0.4)	.653
Major surgery	93 (21.2)	56 (19.8)	.640
Major illness	53 (12.1)	38 (13.4)	.600
Having do-not-resuscitate order	21 (4.8)	2 (0.7)	**.002**
**Clinical characteristics, (mean ± SD)**			
Temperature (^o^C)	37.6 ± 1.1	37.6 ± 1.1	.739
Heart rate (/min)	96.6 ± 19.7	94.9 ± 20.3	.259
Respiration rate (/min)	20.1 ± 2.3	20.0 ± 2.2	.385
Oxygen saturation (%)	96.7 ± 2.1	96.9 ± 2.1	.186
Mean arterial blood pressure (mm Hg)	95.8 ± 18.0	97.9 ± 18.1	.127
**Time variable, (Median, IQR)**			
Time from triage to suggestion to transfer (h)	21.8 (15.5-40.5)	17.7 (9.4-26.9)	**<.001 **
ED length of stay (d)	2.8 (1.8-3.9)	0.9 (0.6-1.5)	**<.001 **

Abbreviations: CCI, Charlson Comorbidity Index; CAD, coronary artery disease; IQR: Interquartile range; SD, standard deviation; ED, emergency department. ^a^ *P* value was examined by chi-square test or *t* test or Mann-Whitney test to compare the two groups.

 After propensity score matching, there were 225 patients who were not transferred matched to 225 patients who were transferred. The presence of a DNR order was more prevalent among those who were not transferred (2.7% vs 0.0%; *P* = .043). Those not transferred had a lower mean arterial blood pressure in triage (93.9 ± 18 vs 98.0 ± 18.6 mm Hg; *P* = .024) and had fewer examinations after suggestion to transfer (43.1% vs 53.3%; *P* = .034) than patients who were transferred.

 The non-transfer group had a longer time from triage to suggestion to transfer (median 19.8 vs 18.2 hours, *P* = .041) and a longer ED length of stay (median 2.7 vs 0.9 days, *P* < .001) compared with those who were transferred. There was no difference in discharge diagnosis and total length of stay among those receiving transfer vs. those who did not. In-hospital mortality was low for both groups (3.1% in the medical center vs 1.3% in regional hospitals; *P* = .344). The results of the matching and analysis are displayed in Tables 2 and 3.

**Table 2:** Demographic Features, Medical Histories, Clinical Characteristics, and Time of Transfer of Patients Transferred and Not Transferred to Regional Hospitals After Propensity Score Matching

**Variables**	**Not Transferred (n = 225) **	**Transferred (n = 225)**	***P*** **Value** ^b^
**Demographic variables, No. (%)**			
Age (≥65 y)	99 (44.0)	102 (45.3)	.831
Gender (male)	121 (53.8)	125 (55.6)	.776
Education (above senior high school)	132 (58.7)	130 (57.8)	.920
Residential area			.078
Taipei city	92 (40.9)	110 (48.9)	
New Taipei city	117 (52.0)	103 (45.8)	
Other	16 (7.1)	12 (5.3)	
CCI, (Mean ± SD)	1.4 ± 1.8	1.3 ± 1.9	.783
**Medical histories not included in CCI, No. (%)**			
CAD/valvular heart disease/arrhythmia	44 (19.6)	45 (20.0)	1.000
Bedridden	8 (3.6)	4 (1.8)	.391
Tracheostomy	0 (0.0)	1 (0.4)	1.000
Major surgery	43 (19.1)	44 (9.6)	1.000
Major illness	29 (12.9)	29 (12.9)	1.000
Having do-not-resuscitate order	6 (2.7)	0 (0.0)	**.043 **
**Clinical characteristics**			
Temperature (^o^C), (SD)	37.6 ± 1.1	37.6 ± 1.1	.990
Heart rate (/min), (SD)	95.9 ± 20.1	94.2 ± 20.7	.376
Respiration rate (/min), (SD)	20 ± 2.4	19.9 ± 2.3	.711
Oxygen saturation (%), (SD)	96.6 ± 2.1	98.9 ± 2.1	.283
Mean arterial blood pressure (mm Hg), (SD)	93.9 ± 18.0	98 ± 18.6	**.024**
Discharge diagnosis, No. (%)			
1^st^: Pneumonia	47 (20.9)	41 (18.2)	.531
2^nd^: Urinary tract infection	40 (17.8)	40 (17.8)	1.000
3^rd^: Cellulitis	24 (10.7)	30 (13.3)	.424
4^th^: Cerebral vascular disease	22 (9.8)	20 (8.9)	.857
5^th^: Acute cholecystitis	13 (5.8)	9 (4.0)	.521
Examinations after suggestion to transfer,^a^ No. (%)	97 (43.1)	120 (53.3)	**.034 **
Diagnosis change, n (%)	8 (3.6)	17 (7.6)	.072
**Time variable, (Median, IQR)**			
Time from triage to suggestion to transfer (h)	19.8 (13.7-35.5)	18.2 (10.1-31.0)	**.041**
ED length of stay (d)	2.7 (1.8-3.9)	0.9 (0.6-1.6)	**<.001 **

Abbreviations: CCI, Charlson Comorbidity Index; CAD, coronary artery disease; IQR: Interquartile range; SD, standard deviation; ED, emergency department. ^a^ Examinations after suggestion to transfer means that the patients received additional medical examinations after transfer decision was made, such as ultrasonography, computed tomography, and endoscopy. ^b^ *P* value was examined by McNemar test or paired *t* test or Wilcoxon signed rank test to compare two groups.

**Table 3:** Outcome for Transferred and Not Transferred Patients

	**Not Transferred (n = 225)**	**Transferred (n=225)**	***P*** **Value** ^b^	**95% CI** ^c^ ** for the Between-Group Difference**
Hospital length of stay (days), (IQR)	6 (0-11)	7 (4-11)	.108	-2.54 to 0.81
Total length of stay (days),^a^ (IQR)	8.7 (4.7-13.9)	7.9 (5.7-11.7)	.245	-0.77 to 2.61
In-hospital mortality, No. (%)	7 (3.1)	3 (1.3)	.344	-0.9% to 4.5%

Abbreviation: IQR: Interquartile range. ^a^ Total length of stay were the sum of the time spent in hospitalization and ED stay. ^b^ *P* value was examined by McNemar test or Wilcoxon signed rank test to compare two groups. ^c^ Confidence interval for the between-group difference (not transferred group – transferred group).

## Discussion

 This study compared length of stay and mortality between patients who accepted or refused transfer from the medical center after primary treatment, and this study supported our hypothesis of similar length of stay outcome between the two groups in this bed-to-bed transfer program. The mortality rates were low for both groups and our sample may be too small to compare the difference. In addition, we found a shorter time from initial triage to suggestion to transfer, and younger patients with stable vital signs were related to a higher transfer acceptance rate. On the other hand, having a DNR order was more prevalent among those who were not transferred. The possible reasons included that these patients may have a longer term relationship with primary care doctors in the medical center. This phenomenon is congruent with previous studies of medical resource utilization of patients in Taiwan.^31,32^ We also found that the examinations after suggestion to transfer and ED length of stay were different between the two groups. More examinations were performed in regional hospital probably because transferred patients were new to them. The prolonged ED length of stay in the non-transfer group was partly because some patients (n = 127) completed treatment in the ED and were discharged. This reflected the problem of ED overcrowding and boarding of patients, underscoring the importance of this bed-to-bed transfer program.

 Importantly, the results showed no significant differences in the total length of stay among those receiving transfer vs those who did not in this study; this was reflected in the previous studies that examined patients with medical or surgical problems who were transferred^14,33,34^ but was contradicted by others.^17,35^ The reason that there was no significant difference in total length of stay among patients might be because this program focused on a transfer of patients with common medical problems to regional hospital model. Rush et al found that patients who were severely ill with sepsis and on a ventilator had longer total length of stay after transfer.^16^ Salehi et al also proposed that prolonged emergency stay time would lead to an increase in the length of hospitalization due to several contributing factors such as age, comorbidities, and subspecialist ward requirement.^1^ The finding in this study is different from the previously mentioned studies because our patient population was younger, with lower CCI, and only needed general wards,^1^ therefore, there may be no significant difference in the length of stay after hospitalization. Patients in the bed-to-bed program could be admitted directly into regional hospitals, while those who refused and stayed in the ED of medical centers maintained their current treatment.^15,36^ The ED length of stay was significantly shorter and was reduced by 1 day in the transferred group in our study. This outcome supports that one goal of the bed-to-bed program—early hospitalization—actually reduced ED length of stay but did not influence total length of stay. The results in this study show that, in the case of ED crowding, referral could reduce emergency stay time, and would not increase the total length of stay. Therefore, the referral program can provide improvements in ED crowding.

 Whether there is a different mortality rate in different medical units is always of great concern. With the caveat of an underpowered sample, we did not find statistically significant in-hospital mortality differences between the two groups. This is congruent with previous studies of the transfer of patients with surgical problems.^14,16^ This result shows that transferring patients from medical centers to regional hospitals does not increase mortality risk. However, patients transferred to academic or large hospitals had significantly reduced mortality in another study.^17^ One possible reason for this is that the subject of the previous study was focused on patients with a history of stroke receiving some form of active stroke intervention (eg, intravenous tissue plasminogen activator, thrombectomy).^17^ This is a critically ill group who should be treated and given further care in medical centers. This also highlights the importance of providing healthcare in appropriate hospital levels according to the severity of illness and diagnosis. Our study included the transfer of patients with urgent and less urgent problems to regional hospitals by assessing TTAS.

 Our study found that the bed-to-bed transfer program showed no difference in total length of stay and could decrease ED length of stay. We can extrapolate from this model to address the same ED crowding problem in other cities. Therefore, when medical teams evaluate the feasibility of transferring patients to regional hospitals, it is suggested that they develop more standardized assessment criteria for transfer and for decision-making by the medical team to reduce possible mistakes. As shown in our results, common diseases are suitable for treatment in community hospitals (eg, pneumonia, urinary tract infection, cellulitis). Furthermore, the reliability and validity of the screening tool should be developed and examined for selecting patients suitable for transfer.

###  Limitations

 Although this study addressed the important findings, there are some limitations to consider. First, this study may be underpowered to detect differences in mortality. Therefore, a larger multi-center randomized study would be the next step to confirm our findings. Second, we used propensity score matching to reduce the effect of confounding factors. However, we did not have more granular data (ie, symptoms, initial inpatient management) that could have been controlled. Third, this retrospective cohort study included only one medical center. The results may not be generalized to all individuals needing a transfer.

## Conclusion

 This program had a 24% transfer rate and this propensity score-matched cohort study found that the program resulted in similar total length of stay between patients who were transferred and those who were not transferred. The study may be underpowered to detect differences in mortality because of a relatively small sample size. Therefore, this healthcare delivery model may be useful in other cities facing similar problems of ED crowding.

## Ethical issues

 This project was approved by The National Taiwan University Hospital Research Ethics Committee (IRB No.: 201806084RIND).

## Competing interests

 Authors declare that they have no competing interests.

## Authors’ contributions

 NWK, CHH, and CCF initiated the study. NWK designed the study, conducted the interviews and prepared the original drafts of the manuscript. CLT and SCC assisted with the design. CLT, CHH, and SCS assisted with the data analysis. CLT and SCC trained and supported NWK in the data collection and analysis methods. All authors made significant contributions to the final manuscript. NWK and SCS are guarantors.

## Funding

 This work was supported by The National Taiwan University Hospital project grant 108-004140.

## Supplementary files

Supplementary file 1 contains Tables S1 and S2.
