# Supplementary material for: Bed-to-Bed Transfer Program Among Patients Who Need Hospitalization in a Crowded Emergency Department in Taiwan
Source: Int J Health Policy Manag. 2021 Aug 24;11(9):1844–51. doi: 10.34172/ijhpm.2021.88 (PMC9808224; doi:10.34172/ijhpm.2021.88)
Supplement: Supplementary file 1 — contains Tables S1 and S2. [file ijhpm-11-1844-s001.pdf]

Article title: Bed-to-Bed Transfer Program Among Patients Who Need Hospitalization in a Crowded Emergency Department in Taiwan

Journal name: International Journal of Health Policy and Management (IJHPM)

Authors' information: Nai-Wen Ku[1,2], Chu-Lin Tsai[3,2], Shyr-Chyr Chen[3,2], Chien-Hua Huang[3,2], Cheng-Chung Fang[3,2], ShiowChing Shun[4]*

[1]School of Nursing, National Taiwan University, Taipei, Taiwan.

[2]Department of Emergency, National Taiwan University Hospital, Taipei, Taiwan.

[3]Department of Emergency Medicine, College of Medicine, National Taiwan University, Taipei, Taiwan.

[4]Institute of Clinical Nursing, College of Nursing, National Yang Ming Chiao Tung University, Taipei, Taiwan.

(*Corresponding authors: scshun@nycu.edu.tw)

Supplementary file 1

Table S1. Multivariate logistic regression model analysis for predicting transfer (n=721)

**Variable**	Coefficient	Waldχ2	Odds Ratio	95% C.I.[a]	*P* Value[b]
Sex	0.068	0.177	1.071	0.779~1.473	.674
Age	-0.015	11.480	0.985	0.976~0.994	**.001**
CCI	0.027	0.321	1.027	0.935~1.129	.577
**Triage levels**					
Level 1(N0=0, Yes=1)	-0.680	0.189	0.507	0.024~10.837	.664
Level 2(N0=0, Yes=1)	-1.028	1.537	0.358	0.070~1.817	.358
Level 3(N0=0, Yes=1)	-0.843	1.096	0.430	0.089~2.086	.295
**Initial Diagnosis**					
Pneumonia (N0=0, Yes=1)	-0.159	0.469	0.853	0.541~1.345	.494
Urinary tract infection (N0=0, Yes=1)	0.074	0.106	1.077	0.689~1.684	.745
Cellulitis (N0=0, Yes=1)	-0.597	5.097	0.550	0.328~0.925	**.024**
Cerebral vascular disease (N0=0, Yes=1)	0.378	1.589	1.460	0.811~2.630	.207
Acute cholecystitis (N0=0, Yes=1)	0.017	0.002	1.017	0.466~2.221	.966
Cancer population (N0=0, Yes=1)	0.778	0.942	2.177	0.453~10.476	.332
Time from triage to transfer suggestion (hours)	-0.016	16.074	0.984	0.976~0.992	**<.001**

[a]C.I.: Confidence Interval

[b]*P* value was examined by Logistic regression analysis

Table S2. Demographics comparing patients whose referral sheets were replied vs. those whose referral sheets were not replied.

	Referral sheets replied (n=283)	No referral sheets replied (n=137)	*P* Value[b]
**Demographic variables, n (%)**			
Age (≥ 65 years)	58.9 (19.7)	55.1 (20.2)	.061
Sex (male)	152 (52.7)	66 (48.2)	.342
Education (Above senior high school)	173 (0.6)	84 (0.6)	.965
Residential area			.701
Taipei city	135 (47.7)	71 (51.8)	
New Taipei city	148 (45.9)	57 (41.6)	
Other	18 (6.4)	9 (6.6)	
Charlson Comorbidity Index, (M±SD)	1.3±1.9	1.1±1.8	.253
**Medical histories not included in Charlson Comorbidity Index, n (%)**			
CAD[a]/Valvular heart disease/Arrhythmia	51 (18)	18 (13.1)	.258
Bedridden	4 (1.4)	4 (2.9)	.510
Tracheostomy	1 (0.4)	0 (0)	1.000
Major surgery	56 (19.8)	19 (13.9)	.182
Major illness	38 (13.4)	16 (11.7)	.727
Having do-not-resuscitate order	2 (0.7)	5 (3.6)	.074

[a]CAD: coronary artery disease

[b]*P* value was examined by Chi-square test or *t* test or Mann-Whitney test to compare the two groups
